# Prevalence and determinants of anemia among pregnant women in Ethiopia; a systematic review and meta-analysis

**DOI:** 10.1186/s12878-017-0090-z

**Published:** 2017-10-17

**Authors:** Getachew Mullu Kassa, Achenef Asmamaw Muche, Abadi Kidanemariam Berhe, Gedefaw Abeje Fekadu

**Affiliations:** 1 College of health Sciences, Debre Markos University, Debre Markos, Ethiopia; 2 Department of Epidemiology and Biostatistics, Institute of Public Health, University of Gondar, Gondar, Ethiopia; 3 Department of Nursing, College of Medicine and Health Science, Adigrat University, Tigray, Ethiopia; 4 School of Public Health, College of Medicine and Health Sciences, Bahir Dar University, P.O.Box 79, Bahir Dar, Ethiopia

**Keywords:** Prevalence of anemia, Anemia during pregnancy, Short birth interval, Malaria during pregnancy, Ethiopia, Meta-analysis, Systematic review

## Abstract

**Background:**

Anemia during pregnancy is one of the most common indirect obstetric cause of maternal mortality in developing countries. It is responsible for poor maternal and fetal outcomes. A limited number of studies were conducted on anemia during pregnancy in Ethiopia, and they present inconsistent findings. Therefore, this review was undertaken to summarize the findings conducted in several parts of the country and present the national level of anemia among pregnant women in Ethiopia.

**Methods:**

Preferred Reporting Items for Systematic Reviews and Meta-Analyses (PRISMA) guideline was followed for this systematic review and meta-analysis. The databases used were; PUBMED, Cochrane Library, Google Scholar, CINAHL, and African Journals Online. Search terms used were; anemia, pregnancy related anemia and Ethiopia. Joanna Briggs Institute Meta-Analysis of Statistics Assessment and Review Instrument (JBI-MAStARI) was used for critical appraisal of studies. The meta-analysis was conducted using STATA 14 software. The pooled Meta logistic regression was computed to present the pooled prevalence and relative risks (RRs) of the determinate factors with 95% confidence interval (CI).

**Results:**

Twenty studies were included in the meta-analysis with a total of 10, 281 pregnant women. The pooled prevalence of anemia among pregnant women in Ethiopia was 31.66% (95% CI (26.20, 37.11)). Based on the pooled prevalence of the subgroup analysis result, the lowest prevalence of anemia among pregnant women was observed in Amhara region, 15.89% (95% CI (8.82, 22.96)) and the highest prevalence was in Somali region, 56.80% (95% CI (52.76, 60.84)). Primigravid (RR: 0.61 (95% CI: 0.53, 0.71)) and urban women (RR: 0.73 (95% CI: 0.60, 0.88)) were less likely to develop anemia. On the other hand, mothers with short pregnancy interval (RR: 2.14 (95% CI: 1.67, 2.74)) and malaria infection during pregnancy (RR: 1.94 (95% CI: 1.33, 2.82)) had higher risk to develop anemia.

**Conclusions:**

Almost one-third of pregnant women in Ethiopia were anemic. Statistically significant association was observed between anemia during pregnancy and residence, gravidity, pregnancy interval, and malaria infection during pregnancy. Regions with higher anemia prevalence among pregnant women should be given due emphasis. The concerned body should intervene on the identified factors to reduce the high prevalence of anemia among pregnant women.

**Electronic supplementary material:**

The online version of this article (10.1186/s12878-017-0090-z) contains supplementary material, which is available to authorized users.

## Background

World health organization (WHO) defines anemia as a low blood hemoglobin concentration. It is one of the major public health problems globally with diverse consequences [1, 2]. It affects the physical health and cognitive development of individual causing low productivity and poor economic development of a country [1, 3]. The problem is also related to high maternal and infant morbidity and mortality especially in developing countries [4, 5].

WHO report showed that anemia affects more than half a billion reproductive age women globally. From this, 38% of the anemic women were pregnant [5]. Anemia is the most common complication related to pregnancy, which affects almost half of pregnant women globally [6–10]. It usually results due to the normal physiological changes of pregnancy resulting in hemoglobin concentration [6, 11]. The problem is more common in developing countries where there is inadequate diet and poor prenatal vitamins and iron and folic acid intake [1, 6, 12]. The most common type of anemia is iron deficiency anemia which mainly affects women of reproductive age group, particularly pregnant women [4, 13].

Several studies have shown that anemia during pregnancy has several adverse effects. Based on the type and severity of anemia, the pregnancy may have poor maternal and fetal outcomes. The most common obstetric problems of anemia include; abortion, prematurity, intrauterine fetal death, low birth weight and perinatal mortality [4, 6, 14–16].

Even though studies have been conducted on the magnitude of anemia among pregnant women in Ethiopia, they present inconsistent and inconclusive findings. So, this systematic review and meta-analysis was conducted to determine the prevalence and determinants of anemia among pregnant women in Ethiopia using the available published evidence. The study will be important to design appropriate interventions to reduce the high burden of the disease.

## Methods

### Study design and search strategy

A systematic review of published studies was used to determine the prevalence of anemia and its determinant factors among pregnant women in Ethiopia. Review of all published studies was done in the following major databases; PubMed, Cochrane Library, Google Scholar, CINAHL, and African Journals Online. The search for published studies was not restricted by time, and all published articles up to January 01/2017 were included into the review. Search of the reference list of already identified studies to retrieve additional articles was done. The search terms used were; “anemia OR anemia during pregnancy OR determinants of anemia AND Ethiopia”. Preferred Reporting Items for Systematic Reviews and Meta-Analyses (PRISMA) guideline was strictly followed when conducting this review [17].

### Study selection and eligibility criteria

This review included studies that were conducted and published on anemia among pregnant women in Ethiopia. All studies conducted at the community or health institution level were included. Studies that provide the prevalence of anemia in pregnant women using the WHO definition (hemoglobin level less than 11 g/dl), and published in the English language were included. Studies conducted among pregnant women but who had comorbidities like; like HIV/AIDS, renal disease, and other medical or surgical conditions were excluded from this study. Articles were assessed for inclusion using their title, abstract and then a full review of papers was done before inclusion to the final review.

### Outcome of interest

The primary outcome of this study was magnitude of anemia during pregnancy. The WHO defines anemia in pregnany as low blood hemoglobin concentration, below 11 g/dl or hematocrit level less than 33% [1]. The determinant variables included in this review were; residence (urban vs rural), pregnancy interval (less than two years, greater than or equal to two years), malaria infection during pregnancy and total number of pregnancy (primigravida or multigravida). Primigravida refers to women who are pregnant for the first time and multigravida refers to women who are pregnant two or more times [18, 19].

### Quality assessment and data collection

Joanna Briggs Institute Meta-Analysis of Statistics Assessment and Review Instrument (JBI-MAStARI) was used for critical appraisal of studies [20]. Two reviewers independently assessed the articles for overall study quality and for inclusion in the review. Any unlear information and disagreement which arises between the reviewers was resolved through discussion and by involving a third reviewer. The researchers developed a data extraction tool. The tool included information on the name of the author/s, publication year, study period, study design, sample size, study area, age of study participants, response rate, mean hemoglobin level, and the prevalence of anemia. Inaddition, the tool contains questions on the prevalence of anemia by residence, number of pregnancy, malaria infection during pregnancy and pregnancy gap.

### Publication bias and heterogeneity

Publication bias and heterogeneity were assessed using the Egger’s and Begg’s tests [21, 22]. A *p*-value less than 0.05 were used to declare statistical significance of publication bias. The heterogeneity of studies was also checked using *I* ^*2*^ test statistics. The *I* ^*2*^ test statistics of 25%, 50%, and 75% was declared as low, moderate and high heterogeneity respectively [23]. A p-value less than 0.05 was used to declare heterogeneity. For the test result which indicates the presence of heterogeneity, random effect model was used as a method of analysis, since it reduces the heterogeneity of studies [23].

### Statistical methods and analysis

Data were entered into Microsoft Excel and then exported to STATA 14 software for further analysis. Forest plot was used to present the combined estimate with 95% confidence interval (CI) of the meta-analysis. Subgroup analysis was conducted by regions of the country and type of study design. The effect of selected predictor variables which include; number of pregnancy, malaria infection during pregnancy, pregnancy gap, and residence on the anemia during pregnancy was analyzed using separate categories of meta-analysis. The findings of meta-analysis were presented using forest plot and relative risk (RR) with its 95% CI.

## Results

### Study selection

**Fig. 1:**
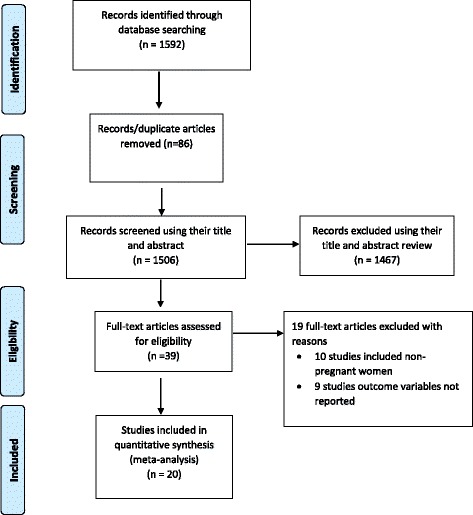
Flow diagram of the studies included in the Meta-analysis

This review included published studies on anemia among pregnant women in Ethiopia. The electronic search was done on several databases, which include; PUBMED, Cochrane Library, Google Scholar, CINAHL, and African Journals Online. The review found a total of 1592 published articles. From this, 86 duplicate records were removed and 1467 records were excluded after screening by title and abstracts. A total of 39 full-text articles were screened for eligibility. From this, 19 articles were excluded since they included non-pregnant women and the outcome variables was not reported. Finally, 20 studies were included in the final quantitative meta-analysis (Fig. 1).

### Characteristics of included studies

All included studies were cross-sectional conducted among pregnant women. The minimum sample size was 150 participants in a study conducted in Nekemte [24]. While, the higher sample size was 1678, conducted in Haramaya district of Oromia region [25]. Overall, this meta-analysis included a total of 10, 281 pregnant women. All studies used the WHO definition of anemia during pregnancy [1]. The minimum and maximum age of pregnant women included in this review were 14 years and 42 years respectively. Thirteen, 65% of the included studies were conducted at health institution [24, 26–37] and 7(35%) of studies were community-based studies [25, 38–43]. Most of the regions in Ethiopia were represented in this review. One of the study was conducted in Addis Ababa, capital city of Ethiopia [34], 3 were from Amhara region [27, 28, 36], 6 from Oromia region [24, 25, 30, 35, 37, 42], 1 from Somali region [38], 5 from SNNPR [29, 32, 33, 40, 43], 2 from Tigray region [26, 31], and 2 were nationwide studies [39, 41] (Table 1).

**Table 1:** Summary characteristics of included studies in the meta-analysis

Author, year of publication	Study area	Study year	Type of cross sectional study	Sample size	Response rate (%)	Mean hemoglobin level (in g/dl)	Prevalence of anemia among pregnant women
Alene KA. and Dohe AM., 2014 [38]	Gode town, Somali Region	2013	Community based	581	99.3	10.79	56.8
Abriha A. et al., 2014 [26]	Mekele town, Tigray Region	2014	Facility based	632	97.9	11.7	19.7
Alem M. et al., 2013 [27]	Azezo Health Center, Gondar, Amhara Region	2011	Facility based	384	100	–	21.6
Alemu T. & Umeta M., 2015 [39]	Data from 2011 EDHS	2011	Community based	1212	–	–	23
Ayenew F. et al., 2014 [28]	Debre Birhan, Amhara region	2013	Facility based	330	89.4	–	9.7
Bekele A.et al., 2016 [29]	Arba Minch, SNNPR	2015	Facility based	332	100	–	32.8
Ejeta E. et al., 2014 [30]	Nekemte Referral Hospital, Oromia Region	2014	Facility based	286	100	12.67	29
Gebre A. & Mulugeta A., 2015 [31]	Northwestern zone, Tigray Region	2014	Facility based	714	97.7	11.21	36.1
Gebremedhin S. et al., 2014 [41]	Eight rural woredas of Tigray, Amhara, Oromia and SNNP regions	2012	Community based	445	93	11.5	33.2
Gebremedhin S, Enquselassie F., & Umeta M., 2014 [40]	Sidama, SNNPR	2011	Community based	700	93.1	11.4	31.6
Gedefaw L.et al., 2015 [32]	Wolayita Soddo Otona Hospital, SNNPR	2014	Facility based	363	100	11.55	39.9
Getachew M. et al., 2012 [42]	Districts around Gilgel Gibe Dam area, Oromia Region	2011	Community based	388	98.7	10.9	53.9
Gies S. et al., 2003 [33]	Awassa, SNNPR	2001	Facility based	403	100	12.3	15.1
Jufar AH. & Zewde T., 2014 [34]	Tikur Anbesa Specialized Hospital, Addis Ababa	2013	Facility based	395	100	12	21.3
Kedir H.et al., 2013 [25]	Haramaya district, Oromia Region	2010	Community based	1678	94.7	11	43.9
Kefiyalew F. et al., 2014 [35]	Bisidimo Hospital, Babile Woreda, Somalie	2013	Facility based	258	100	11.4	27.9
Melku M.et al., 2014 [36]	Gondar University hospital, Amhara region	2012	Facility based	302	100	11.96	16.6
Mihiretie H. et al., 2015 [24]	Nekemte, Oromia region	2011	Facility based	150	100	–	52
Nega D.et al., 2015 [43]	Arba Minch Town, SNNPR region	2013	Community based	354	96.3	11.73	34.6
Obse N.et al., 2013 [37]	Shara woreda, West Arsi zone, Oromia region	2011	Facility based	374	100	12.05	36.6

### Prevalence of anemia among pregnant women

**Fig. 2:**
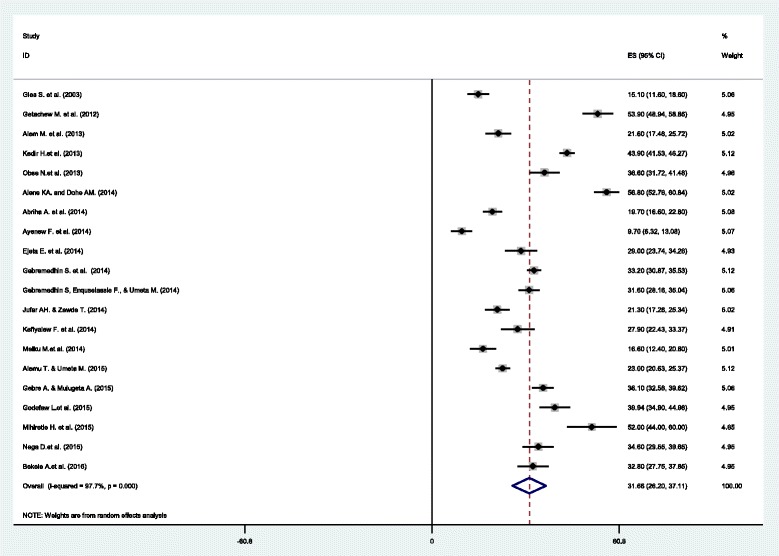
Forest plot displaying the pooled prevalence of anemia among pregnant women in Ethiopia

The minimum prevalence of anemia was 9.7% observed in a study conducted in North Shoa zone [28]. The highest, 56.8% was observed in a study conducted in Eastern Ethiopia [38]. The *I* ^*2*^ test result showed high heterogeneity (I^2^ 97.7%, p = <0.001). Using the random effect analysis, the pooled prevalence of anemia among pregnant women in Ethiopia was 31.66% (95% CI (26.20, 37.11)) (Fig. 2).

A subgroup analysis by region in Ethiopia was computed to compare the prevalence of anemia acroos different participants characterstics. Based on this analysis, the lowest prevalence of anemia among pregnant women was observed in Amhara region, 15.89% (95% CI (8.82, 22.96)) and the highest prevalence was in Somali region; 56.80%(95% CI (52.76, 60.84)). A higher prevalence (39.49%) of anemia among pregnant women was observed in studies conducted at community level than facility based studies (27.31%) (Table 2).

**Table 2:** Sub-group analysis of prevalence of anemia among pregnant women in Ethiopia

Sub group	No. of included studies	Prevalence (95% CI)	Heterogeneity statistics	*p*-value	*I* ^*2*^
By region					
Addis Ababa City	1	21.30 (17.26, 25.34)	–	–	–
Amhara region	3	15.89 (08.82, 22.96)	19.89	<0.001	89.9
Oromia region	6	40.44 (32.67, 48.20)	83.85	<0.001	94.0
SNNPR	5	30.71 (21.74, 39.68)	87.75	<0.001	95.0
Somali region	1	56.80 (52.76, 60.84)	–	–	–
Tigray region	2	27.88 (11.81, 43.95)	46.91	<0.001	97.9
Nationwide study	2	28.10 (18.11, 38.10)	36.12	<0.001	97.2
By study type					
Community based study	7	39.49 (30.84, 48.14)	321.93	<0.001	98.1
Institutional based study	13	27.31 (21.51, 33.10)	289.66	<0.001	97.7

### Association of malaria infection and anemia during pregnancy

**Fig. 3:**
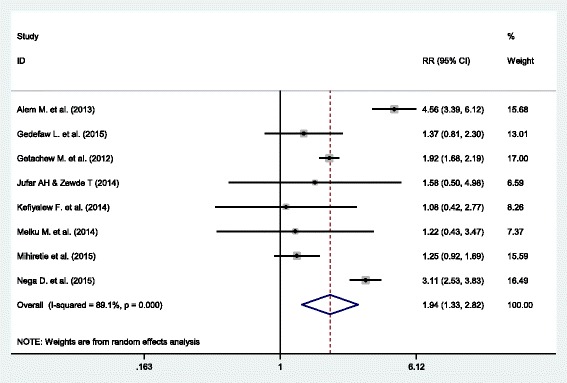
Forest plot displaying the effect malaria attack and anemia among pregnant women in Ethiopia

Women who had malaria infection during pregnancy were almost two times more likely to develop anemia during pregnancy than women had no such infection, RR: 1.94 (95% CI (1.33, 2.82)). The heterogeneity test showed statistical evidence of heterogeneity, *p* = <0.001. As a result, weights were calculated using the random-effects analysis. The Begg’s and Egger’s test for publication bias showed no statistical evidence of publication bias, *p*-value = >0.05 and *p*-value = 0.543 respectively (Fig. 3).

### Association of number of pregnancy with anemia during pregnancy

The meta-analysis showed that premigravida women were 61% less likely to develop anemia during pregnancy compared tomultigravida women, RR: 0.61 (95% CI (0.53, 0.71)). The heterogeneity test showed no statistical evidence of heterogeneity, *p* = 0.530. The Begg’s and Egger’s test for publication bias also showed no statistical evidence of publication bias, *p*-value = 0.36 and *p*-value = 0.397 respectively (see Additional file 1).

### Association of short pregnancy interval with anemia during pregnancy

Women who had short pregnancy interval were more than two times more likely to develop anemia during the current pregnancy than women who had more than two years pregnancy interval, RR: 2.14 (95% CI (1.67, 2.74)). The heterogeneity test showed no statistical evidence of heterogeneity, *p* = 0.108. The Begg’s and Egger’s test for publication bias also showed no statistical evidence of publication bias, *p*-value = 0.266 and *p*-value = 0.112 respectively (see Additional file 2).

### Association of residence with anemia during pregnancy

Women living in urban areas were 73% less likely to be anemic during pregnancy than women in the rural area, RR: 0.73 (95% CI (0.60, 0.88)). The heterogeneity test showed statistical evidence of heterogeneity, *p* = 0.003. But, the Begg’s and Egger’s test for publication bias showed no statistical evidence of publication bias, *p*-value = 0.602 and *p*-value = 0.581 respectively (**see** Additional file 3).

## Discussion

This review was conducted to determine the pooled prevalence and determinants of anemia among pregnant women in Ethiopia using published studies. Anemia during pregnancy is associated with increased risk of obstetric problems [44]. Studies have shown that anemia is associated with maternal physical and psychological comorbidity, and with an increased risk of perinatal and maternal morbidity and mortality [45–47].

The pooled meta-analysis of this review found that the prevalence of anemia among pregnant women in Ethiopia was 31.66% (95%CI: (26.20, 37.11)). The 2016 Ethiopian demographic and health survey (EDHS) report showed a lower (24%) prevalence of anemia among reproductive-aged women, and 29% among pregnant women [48]. This showed a higher prevalence of anemia among pregnant women than non-pregnant reproductive age women. This could be explained by an extra demand of iron by the pregnant women for fetal growth and development during pregnancy. This report is lower than the current finding. The possible explanation for the difference could be related to the sampling and study period. The EDHS was conducted in a nationally representative sample across all regions of the country, while this study included only few regions of the country. The current review also included studies conducted since 2001.

A meta-analysis on global trend of anemia showed that 38% of pregnant women were anemic in 2011 [3]. The review also showed that 36% prevalence of anemia among pregnant women in East Africa and 22% prevalence in high-income regions [3]. The East African finding is relatively higher than the findings of this review. A possible explanation could be the time difference between the two reviews in which the current review also included recent studies and the difference in the sociodemographic characteristics of participants included in the review.

Subgroup analysis based on the regions of the country showed a lower and higher prevalence of anemia in Amhara region (15.89%) and Somali region (56.8%) respectively. The difference in the prevalence between the regions in Ethiopia could be attributed due to the difference in the sociodemographic, socioeconomic, iron-folic acid intake and the difference in the magnitude of the communicable and non-communicable diseases. The difference in the number of studies included in each category of analysis could also be the reason for the difference.

The result of the meta-analysis showed that primigravida women were 61% less likely to develop anemia during pregnancy compared to multigravida women. This could be because of the effect of repeated pregnancy in depleting the iron store of a pregnant woman [49, 50]. A study conducted in Malaysia also found a higher proportion of anemia (66.7%) among grand multigravida women [51].

A shorter interpregnancy interval increases the risk of adverse obstetric outcomes [52]. Short birth interval is associated with preterm births, low birth weight, stillbirth and early neonatal death [52]. The current study also found that pregnant women with short pregnancy interval were more than two times more likely to develop anemia during the current pregnancy than women who had more than two years pregnancy interval. This could be explained by the effect of repeated and short interpregnancy interval and breastfeeding on the overall physiologic status of the mother. The woman will not get enough time to recover from the depleted nutrients [50]. A systematic review of the effect of birth spacing on the maternal and child nutritional status found that short birth intervals are related to maternal anemia [49]. Similar findings were also observed in a study conducted in Tanzania [53].

Pregnant women living in urban areas are 73% less likely to be anemic during pregnancy than women in the rural area. The difference in the socioeconomic status, educational and occupational status of pregnant women, difference in the health service access between rural and urban areas could be the justification for the difference. Additionally, inadequate counselling by health professionals in resolving the wrong beliefs and myths regarding the iron supplementation could contribute to higher prevalence of anemia among pregnant women in rural areas [54, 55]. A study conducted in India also showed that pregnant women from the rural areas are more likely to develop anemia than women from the urban area [54].

WHO recommends early diagnosis and effective treatment of malaria infections and the use of long-lasting insecticidal nets (LLINS) during pregnancy [56]. The result of this meta-analysis showed that women who had malaria attack during pregnancy are almost two times more likely to develop anemia during pregnancy. A review of studies conducted in Sub-Saharan African countries also found that there is a higher (26%) risk of severe anemia in pregnant women secondary to malaria infection. Malaria infection is responsible for one in ten cases of severe anemia in pregnant women [46]. Similar findings was also observed in a study conducted in Kenya [57].

This review used a comprehensive search strategy and more than one reviewer was involved in each step of the review process. PRISMA guideline was strictly followed during the review process. This review has certain limitations. Studies included were cross-sectional and the outcome variable may be affected by other confounding variables. Some studies included in this review didn’t consider respondents’ residential altitude above sea level to define anemia. Studies have shown that there is an increase in the hemoglobin level when people’s live at high altitude [58, 59]. These limitations could affect the overall prevalence of anemia in the country presented in this review.

## Conclusions

Almost one-third of pregnant women in Ethiopia were anemic. Statistically significant association was observed between anemia during pregnancy and residence, gravidity, pregnancy interval and malaria infection during their pregnancy. Regions with higher anemia prevalence among pregnant women should be given due attention. Further studies should be conducted to better understand the determinant factors in these regions.

The government and non-governmental organizations should focus on strengthening iron and folic acid supplementation for all pregnant women as part of the routine antenatal care. The use of long-lasting insecticidal nets during pregnancy, early diagnosis and appropriate treatment of malaria in pregnant women, and the use of long-acting family planning methods to prevent short pregnancy intervalsis important and should be stengtehend in areas of higher anemia prevalence in Ethiopia. Health extension workers should be involved in the promotion of antenatal follow-ups and community-based awareness programs, especially in rural areas. Further nationwide studies are needed to understand the determinant factors for anemia in pregnant women.

## Additional files

Additional file 1: Forest plot displaying the effect of gravidity in a pregnant woman and anemia among pregnant women in Ethiopia. Description of figure: This figure presents the effect of gravdity on anemia during pregnancy. Multigravida women are more likely to develop anemia during pregnancy than primigravida. (DOCX 18 kb)

Additional file 2: Forest plot displaying the effect of short pregnancy interval and anemia among pregnant women in Ethiopia. Description of figure: This figure presents the effect of short pregnancy interval on anemia during pregnancy. Women who have shorter pregnancy interval are more likely to develop anemia during pregnancy than women with pregnancy interval of more than two years. (DOCX 17 kb)

Additional file 3: Forest plot displaying the effect residence of pregnant woman and anemia among pregnant women in Ethiopia. Description of figure: This figure presents the effect of residence on anemia during pregnancy. Women who are residing in rural areas are more likely to develop anemia during pregnancy than pregnant women in urban areas. (DOCX 18 kb)

## Abbreviations

CI: Confidence Interval
EDHS: Ethiopian Demographic and Health Survey
LLINS: Long-lasting insecticidal nets
RR: Relative Risk
SNNPR: Southern Nations, Nationalities, and Peoples’ Region of Ethiopia
WHO: World Health Organization
