# Supplementary material for: Prevalence and determinants of anemia among pregnant women in Ethiopia; a systematic review and meta-analysis
Source: BMC Hematol. 2017 Oct 17;17:17. doi: 10.1186/s12878-017-0090-z (PMC5646153; doi:10.1186/s12878-017-0090-z)
Supplement: Supplementary file 1 — Forest plot displaying the effect of gravidity in a pregnant woman and anemia among pregnant women in Ethiopia. Description of figure: This figure presents the effect of gravdity on anemia during pregnancy. Multigravida women are more likely to develop anemia during pregnancy than primigravida. (DOCX 18 kb) [file 12878_2017_90_MOESM1_ESM.docx]

Additional file 1**.** Forest plot displaying the effect of gravidity in a pregnant woman and anemia among pregnant women in Ethiopia
