# Supplementary material for: Prevalence and determinants of anemia among pregnant women in Ethiopia; a systematic review and meta-analysis
Source: BMC Hematol. 2017 Oct 17;17:17. doi: 10.1186/s12878-017-0090-z (PMC5646153; doi:10.1186/s12878-017-0090-z)
Supplement: Supplementary file 2 — Forest plot displaying the effect of short pregnancy interval and anemia among pregnant women in Ethiopia. Description of figure: This figure presents the effect of short pregnancy interval on anemia during pregnancy. Women who have shorter pregnancy interval are more likely to develop anemia during pregnancy than women with pregnancy interval of more than two years. (DOCX 17 kb) [file 12878_2017_90_MOESM2_ESM.docx]

Additional file 2**.** Forest plot displaying the effect of short pregnancy interval and anemia among pregnant women in Ethiopia
