# Supplementary material for: Prevalence and determinants of anemia among pregnant women in Ethiopia; a systematic review and meta-analysis
Source: BMC Hematol. 2017 Oct 17;17:17. doi: 10.1186/s12878-017-0090-z (PMC5646153; doi:10.1186/s12878-017-0090-z)
Supplement: Supplementary file 3 — Forest plot displaying the effect residence of pregnant woman and anemia among pregnant women in Ethiopia. Description of figure: This figure presents the effect of residence on anemia during pregnancy. Women who are residing in rural areas are more likely to develop anemia during pregnancy than pregnant women in urban areas. (DOCX 18 kb) [file 12878_2017_90_MOESM3_ESM.docx]

Additional file 3**.** Forest plot displaying the effect residence of pregnant woman and anemia among pregnant women in Ethiopia
